# Ataxia and oculomotor apraxia caused by a large-scale deletion in the senataxin gene

**DOI:** 10.1007/s13353-025-01001-2

**Published:** 2025-08-20

**Authors:** Joanna M. Rusecka, Biruta Kierdaszuk, Iwona Stępniak, Małgorzata Rydzanicz, Piotr Stawiński, Tomasz Gambin, Damian Loska, Magdalena M. Kacprzak, Magdalena Kaliszewska, Dorota Piekutowska-Abramczuk, Anna M. Kamińska, Ewa Pronicka, Ewa Bartnik, Anna Kostera-Pruszczyk, Rafał Płoski, Agnieszka Sobczyńska-Tomaszewska, Katarzyna Tońska

**Affiliations:** 1 Faculty of Biology, Institute of Genetics and Biotechnology, University of Warsaw, Pawińskiego 5a, Warsaw, 02-106 Poland; 2 Maria Skłodowska-Curie Medical Academy, Solidarności 12, Warsaw, 03-411 Poland; 3 MedGen Medical Center, Wiktorii Wiedeńskiej 9a, Warsaw, 02-954 Poland; 4 Department of Neurology, Medical University of Warsaw, ERN EURO-NMD, Banacha 1a, Warsaw, 02-097 Poland; 5 Institute of Psychiatry and Neurology, Jana III Sobieskiego 9, Warsaw, Poland; 6 Department of Medical Genetics, Medical University of Warsaw, Pawińskiego 3C, Warsaw, 02-106 Poland; 7 Institute of Computer Science, Warsaw University of Technology, Nowowiejska 15/19, Warsaw, 00-665 Poland; 8 Department of Medical Genetics, The Children’s Memorial Health Institute, Dzieci Polskich 20, Warsaw, 04-730 Poland

**Keywords:** *SETX*, R-loop, AOA2, Neurodegenerative disorder, WES, CNV, MtDNA

## Abstract

Senataxin, an RNA/DNA helicase, is a key protein providing genome stability and one of the best characterized R-loop-binding factors playing an important role in transcription and DNA repair processes. Pathogenic *SETX* gene variants cause autosomal recessive spinocerebellar ataxia with axonal neuropathy (AOA2, MIM #606002) and autosomal dominant juvenile amyotrophic lateral sclerosis (ALS4, MIM #602433), rare neurodegenerative disorders characterized by juvenile onset of progressive cerebellar ataxia, axonal sensorimotor peripheral neuropathy, combined upper and lower motor neuron symptoms, and increased serum alpha-fetoprotein (AFP; specific for AOA2). We report two cases of adult patients presenting with cerebellar syndrome, scanned speech, and exercise intolerance which started in the second/third decade of life and were followed by muscle weakness and impaired gait coordination. Whole exome sequencing (WES) was performed to analyze single nucleotide and copy number variants. A decreased coverage of a genomic region of around 16 kb on chromosome 9 (chr9:132,295,852–132,311,876), suggesting a deletion encompassing 5 exons of the *SETX* gene (exons 11–15, NM_015046.7) was observed. This homozygous *SETX* (9q34.13) deletion leads to a frame shift and consequently truncation of the helicase domain in the protein. Loss-of-function variants in the *SETX* gene are known to be pathogenic. Statistical analysis of NGS data from the Polish population identified a few heterozygous carriers, suggesting its region-specific origin.

## Introduction

Hereditary ataxias comprise a group of heterogeneous neurological disorders with autosomal dominant, recessive, X-linked, as well as mitochondrial modes of inheritance. Besides ataxia, other most characteristic symptoms include ophthalmoparesis, polyneuropathy, metabolic abnormalities, and cardiologic involvement. Numerous autosomal dominant ataxias have been described with several common features such as onset in early or mid-adult years, gait ataxia, and nystagmus. These symptoms may be accompanied by dementia, parkinsonism, dystonia, tremor, and neuropathy. The majority of dominant ataxias result from an expansion of CAG trinucleotide repeats in numerous genes. Generally, the age of onset is earlier in recessive than in dominant forms. The phenotype may vary even within one family. Depending on the mechanism of pathogenesis, recessive ataxias are mostly classified as related to oxidative stress and mitochondrial dysfunction, impaired DNA repair, metabolic disorders, and others.

The *SETX* gene (9q34.13) encodes senataxin—a 302.8 kDa ATP-dependent helicase [EC 3.6.4.-], a member of the DNA2/NAM7 helicase family, named for its homology to the Sen1 protein in fungi. Senataxin is a nucleoplasmic protein with no evidence of localization to any subnuclear compartments and with only minimal amounts in the cytoplasm in human (Suraweera et al. 2007; Chen et al. 2014). Senataxin is ubiquitously expressed and consists of a strongly conserved C-terminal RNA helicase domain (a superfamily helicase domain I—SF1), followed by a nuclear localization signal (NLS), and an N-terminal domain important for protein–protein interactions (Lavin et al. 2013). The N-terminus may be essential for correct senataxin localization (Chen et al. 2014).

Pathogenic *SETX* variants are responsible for rare neurological disorders like autosomal dominant juvenile amyotrophic lateral sclerosis (ALS4) and autosomal recessive spinocerebellar ataxia with axonal neuropathy 2 (SCAN2) sometimes referred to as ataxia-oculomotor apraxia 2 (AOA2) (Fogel et al. 2006). Oculomotor apraxia is a common but inconsistent finding, present in about 50% of patients (Ichikawa et al. 2013; Nanetti et al. 2013; Szpisjak et al. 2016). Both diseases are characterized by juvenile (2nd or 3rd decade of life) onset with progressive cerebellar ataxia, combined upper and lower motor neuron symptoms, axonal sensorimotor peripheral neuropathy, and increased serum alpha-fetoprotein (in AOA2) (Lynch et al. 2007; Anheim et al. 2009; Gazulla et al. 2009; Nicolaou et al. 2008; Marelli et al. 2016; Algahtani et al. 2018). *SETX* gene variants may also lead to hypogonadism, ovarian failure (Lynch et al. 2007, Anheim et al. 2009, Kinkar 2021), Homer 2021), and presumably male infertility (Catford et al. 2019; Becherel et al. 2019). Moreover, senataxin exerted an inhibitory effect on the transcriptional response to viral infection, which may have implications for neurological disorders characterized by *SETX* variants (Miller et al. 2015). So far, most of the pathogenic *SETX* variants localize in the helicase domain or in the N-terminal putative protein–protein interaction domain (Nanetti et al. 2013, Anheim et al. 2009, Marelli et al. 2016, Algahtani et al. 2018, Miller et al. 2015, Moreira et al. 2004, Chen et al. 2004, Duquette et al. 2005, Fogel and Perlman 2006, Criscuolo et al. 2006, Bernard et al. 2009, Arning et al. 2013). Large deletions are expected to create a premature termination codon and result in an absent or disrupted protein product. Loss-of-function variants in *SETX* are known to be pathogenic (Moreira et al. 2004).

Senataxin is involved in maintaining genomic integrity and stability (Aguilera and Gómez-González, 2008; Aguilera and García-Muse, 2012, 2013; Becherel et al., 2013). It is required for R-loop RNA–DNA hybrid formation (García-Muse and Aguilera, 2016; Grunseich et al., 2018, Hamperl and Cimprich, 2014; Hatchi et al. 2015; Makharashvili et al. 2018; Skourti-Stathaki et al. 2011, Yüce and West 2013, Cohen et al. 2018), transcription termination (Hasanova et al. 2023), fork integrity protection (Alzu et al. 2012), and DNA double-strand breaks (DSBs) repair. It suppresses DNA damage generated by oxidative stress (Cohen et al. 2018) and may be involved in telomeric stability through the regulation of telomere repeat-containing RNA transcription (De Amicis et al*.*, 2011). Senataxin is also one of the best characterized R-loop-binding factors. R-loop levels are under tight control; thus, variants in genes encoding proteins implicated in R-loop resolution lead to devastating human diseases, often involving neurodegeneration (Groh and Gromak 2014), including ataxias, amyotrophic lateral sclerosis, nucleotide expansion disorders (e.g., Friedreich ataxia and fragile X syndrome), and numerous kinds of neoplasms (Groh et al. 2017; Gatti et al. 2023).

Beside affecting neuronal function, senataxin plays an essential role in sex chromosome inactivation and spermatogenesis (Catford et al. 2019). In humans, pathogenic *SETX* variants are associated with hypogonadism, with reports of premature ovarian insufficiency (Lynch et al. 2007; Gazulla et al. 2009; Becherel et al. 2019; Criscuolo et al. 2006; Le Ber et al. 2005). Novel findings in AOA2 patients with *SETX* variants suggest a role for senataxin in human germ cell development and reproductive function, clinically and histologically consistent with the phenotype seen in *Setx*^−/−^ knockout mice (Catford et al. 2019; Becherel et al. 2019). Loss-of-function *SETX* mutations may lead to defective spermatogenesis and sterility in males, both in mice and humans. As a consequence of *SETX* pathogenic variants, male infertility may appear before neurological symptoms (Becherelet al. 2019).

Sen1 is an ATP-dependent 5′ to 3′ RNA:DNA and DNA helicase essential in yeast involved in one of two major transcription termination pathways—the non-canonical Nrd1 complex dependent pathway (Grzechnik et al. 2015). Moreover, Sen1 is critical for mitochondrial function. The deletion of the Pol II interaction domain (N-terminal truncation of AA 1–975) in yeast caused severe loss of mitochondrial DNA, similar to rho0 (*petite*) cells (Sariki et al. 2016). Although human senataxin also interacts with Pol II, so far studies have not confirmed a mitochondrial role and primarily mitochondrial localization of senataxin in mammals. However, variants in this domain were found to cause more severe phenotypes compared to variants inside the helicase domain (Anheim et al. 2009).

Human senataxin shows extensive sequence homology with the *Saccharomyces cerevisiae* protein Sen1 (Moreira et al. 2004; Sariki et al. 2016) although complementation studies have shown that neither the human ortholog of Sen1 (senataxin ORF) nor the human senataxin helicase domain functionally replace the yeast gene. Introduction of variants found in human *SETX* (only in the helicase domain) to homologous positions in the yeast Sen1 led to a transcription termination defect (Chen et al. 2014).

Although human senataxin localizes in the nucleus, its deletion leads to R-loop accumulation in mitochondrial DNA and increased ROS production (Renaudin et al. 2021).

Here, we present two patients with a rare deletion in the *SETX* gene leading to AOA2, accompanied by mtDNA deletions of uncertain origin in one of them.

## Methods

### Subjects and clinical investigations

Patients with ataxia and a spectrum of additional neurological symptoms were referred to Department of Neurology, Medical University of Warsaw (Patient 1) and Institute of Psychiatry and Neurology in Warsaw (Patient 2) for neurological examination. Detailed clinical evaluation was performed together with brain imaging and a laboratory alpha-fetoprotein test. Skeletal muscle histopathologic analysis was performed for Patient 1. Both patients were directed to genetic screening. Reference values for the AFP level differ as the analyses were performed in different laboratories.

### Genetic diagnosis scheme

#### Patient 1

*POLG* (NG_008218.2) pathogenic variants p.Ala467Thr, p.Trp748Ser, p.Gly848Ser, and p.Thr251Ile screening were performed as described in Piekutowska-Abramczuk et al. (2019). *POLG* coding region sequencing was performed as described in Kaliszewska et al. (2015). Basic mitochondrial DNA screening was performed as described in Kierdaszuk et al. (2009).

Whole exome sequencing (WES) was performed on the proband’s DNA using SureSelect Human All Exon V5 (Agilent Technologies, Palo Alto, CA) and paired-end sequenced (2 × 100 bp) on HiSeq 1500 (Illumina, San Diego, CA). Bioinformatics analysis of WES raw data was performed using a previously described pipeline (Płoski et al*.*, 2014). In brief, raw data were analyzed using bcl2fastq software (Illumina) to generate reads in fastq format. After the quality control steps, including adapter trimming and low-quality read removal, reads were aligned to the hg38 reference genome. The mean depth of coverage in the sequenced sample was 86 ×; 95% of the target sequence was covered at least 20 × and 98% min 10 ×.

Identified variants were annotated with functional information, frequency in the population (including gnomAD (http://gnomad.broadinstitute.org/), as well as an in-house database), and known association with clinical phenotypes, based on both ClinVar (https://www.ncbi.nlm.nih.gov/clinvar/) and HGMD (http://www.hgmd.cf.ac.uk) databases.

The HMZDelFinder algorithm (Gambin et al. 2017) was used to search for small, rare copy number variants (CNVs). The presence of the deletion was confirmed by PCR and Sanger sequencing.

#### Patient 2

The test for triplet expansion in the genes causing Friedreich ataxia (FXN) and spinocerebellar ataxia type 2 (ATXN2) was performed in the Institute of Psychiatry and Neurology in Warsaw. Then, the NGS panel of 152 genes correlated with ataxias, hereditary spastic paraplegias, and rare syndromes accompanied by ataxia or spastic paraplegia was applied, and it was followed by WES in Medgen Medical Center.

Whole exome sequencing was performed using Twist Human Core Exome Plus Kit (Twist Bioscience) and sequenced with Illumina technology (100 × depth of mean coverage). Reads were aligned to the hg38 reference genome. The obtained QC value was < 99% for Q30. Alignment and variant calling were performed with an in-house bioinformatics pipeline. The identified variants were annotated using the Ensembl VEP as well as multiple databases, including dbSNP, dbNSFP, GnomAD, ClinVar, and HGMD. The American College of Medical Genetics and Genomics (ACMG) Standards and Guidelines for the interpretation of sequence variants were followed in this study. The XHMMv1.0 algorithm and in-house scripts were used to search for small, rare CNVs.

*SETX* deletion was confirmed by MLPA.

## Results

### Clinical and laboratory findings

#### Patient 1

A 44-year-old female patient with a slightly progressive cerebellar syndrome and weakness of distal muscles was diagnosed in the Department of Neurology, Medical University of Warsaw. The first symptoms appeared around the age of 19. Impaired coordination was quickly accompanied by slurred speech, gaze fixation difficulties, and signs of peripheral polyneuropathy. Neurological examination showed abnormal eye movements, ataxic dysarthria, and cerebellar syndrome in upper and lower limbs. Moreover, atrophy and weakness of the distal muscles in upper and lower limbs and areflexia were present without any sensory deficits. The patient requires a wheelchair mainly due to the cerebellar syndrome. On the SARA scale (Schmitz-Hübsch et al*.*, 2006), she scored 23 points (0—no ataxia, 40—most severe ataxia).

**Fig. 1:**
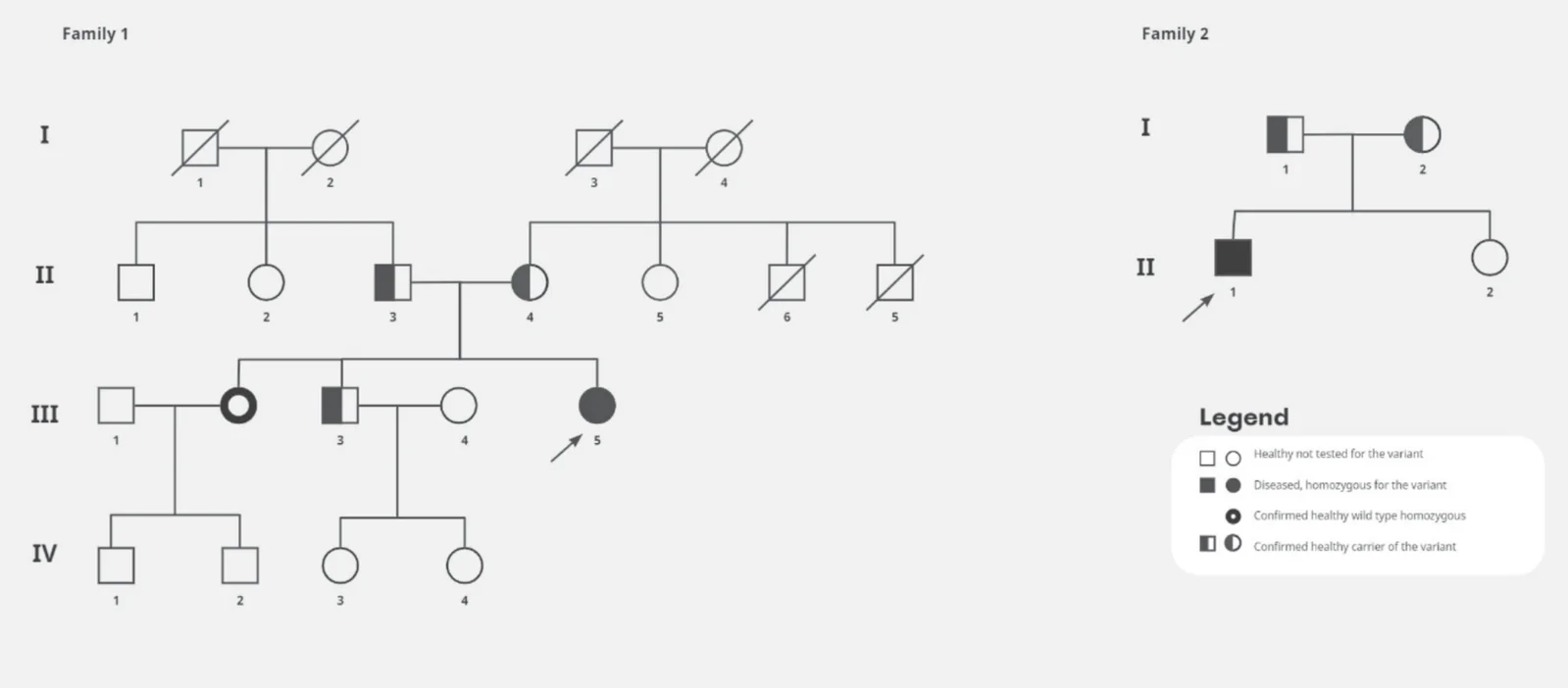
Pedigrees of families

The family history of neurological diseases was negative with no evidence of consanguinity, but the patient’s parents come from villages located close to each other. The pedigree of the family is shown on Fig. 1.

**Fig. 2:**
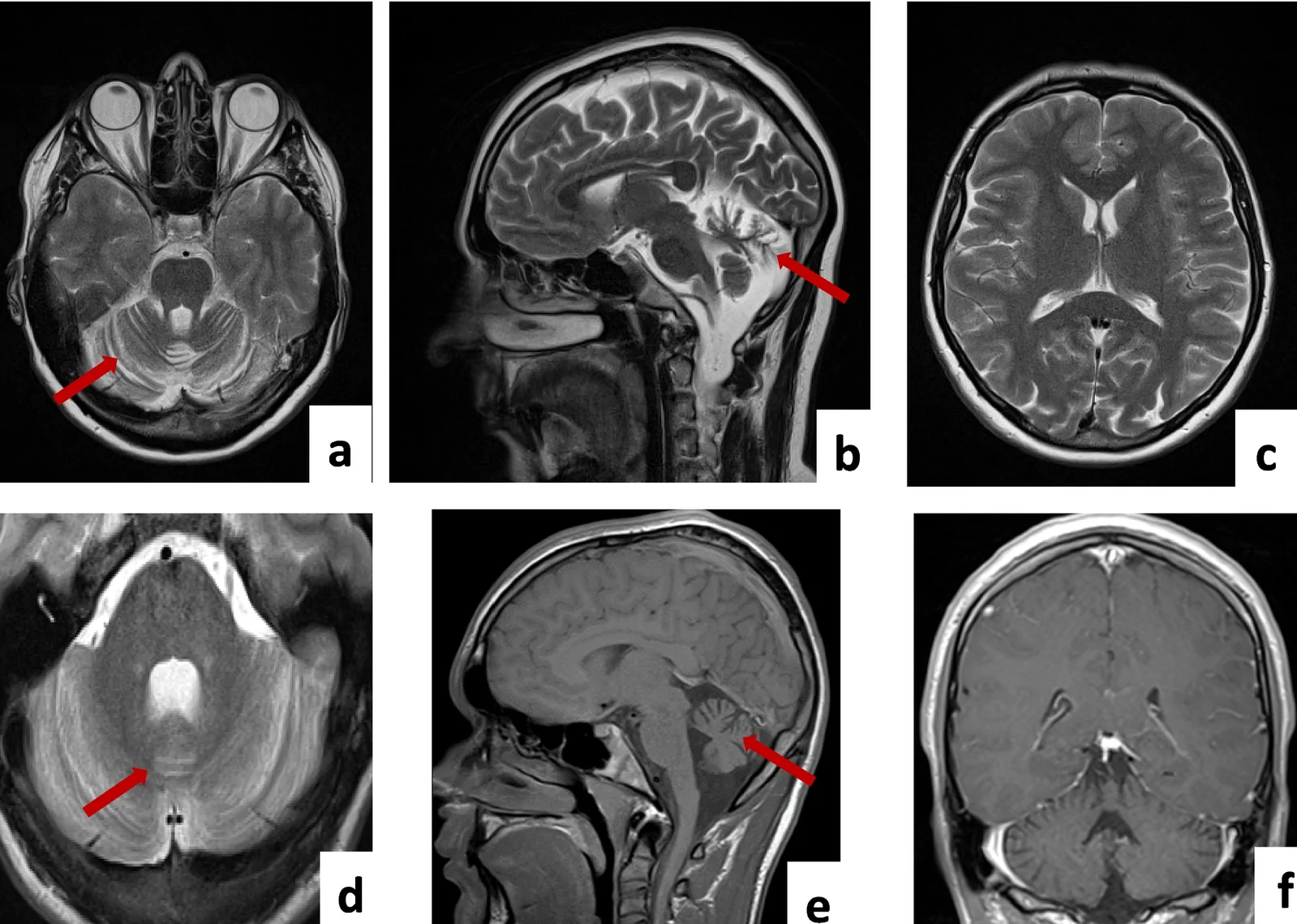
Brain MRI. T2-weighted images, cerebellum atrophy on transverse **a** and sagittal **b** images, and normal supratentorial brain structures **c** of Patient 1. Transverse T2-weighted image **d**, mid-sagittal T1-weighted image shows moderate atrophy of the vermis **e**, coronal T1-weighted image confirms atrophy of the vermis and shows atrophy of the cerebellar hemispheres **f** of Patient 2

**Fig. 3:**
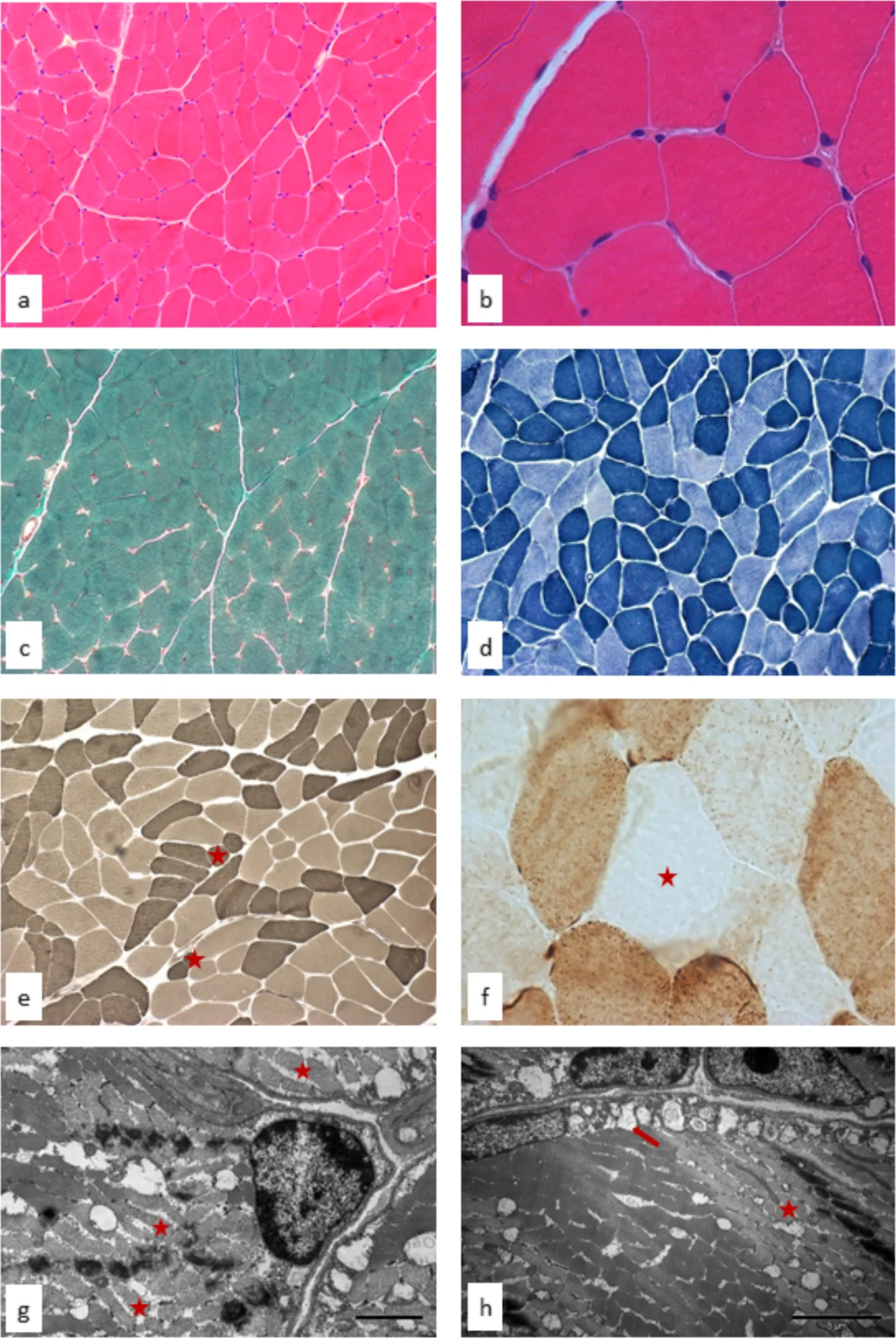
Microscopic analysis of the Patient 1 skeletal muscle biopsy. Hematoxylin and eosin staining × 100 magnification **a** and × 200 magnification **b** shows muscle fibers of various shapes and sizes. Gomori trichrome **c** and NADH **d** staining did not indicate any pathology. On ATPase pH 9.4 staining **e**, atrophy of type 2 muscle fibers is visible (asterisks). COX-negative fibers (asterisk) are present on COX staining **f**. Electron microscopy shows severe myofibril loss **g**, **h** (asterisks) as well as multiple lipid droplets under the sarcolemma **h**, marked with an arrow

Laboratory analysis showed an elevated alpha-fetoprotein (AFP) level—47.1 and 59.8 ng/ml (normal range 0.0–7.0 ng/ml). Nerve conduction studies indicated severe axonal damage of the motor and sensory nerves. Electromyography showed features of acute denervation in the left tibialis anterior muscle. Magnetic resonance imaging (MRI) revealed symmetrical cortical and subcortical cerebellar atrophy; the images were compared to the previous one from 2015 and showed no differences (Fig. 2a–c). In the neuropsychological examination, a weakening of the attention and memory functions with generally correct cognitive functioning of the patient was found. EEG was normal. Cardiological investigation excluded any abnormalities in echocardiography and Holter electrocardiography studies. Morphological analyses of a biopsy of the left biceps brachii muscle presented non-specific myopathic changes with COX-negative fibers. Similarly, on electron microscopy, nonspecific signs of myofibril degeneration were present (Fig. 3).

#### Patient 2

A 17-year-old man was diagnosed in the Institute of Psychiatry and Neurology. He was born after an uncomplicated pregnancy and delivery. Psychomotor development proceeded normally. Gait instability was the first neurological sign and appeared at the age of 10. About 2 years later, choreic movements and strabismus manifested. On neurological examination at age 17 years, oculomotor apraxia, convergent strabismus of the right eye, horizontal nystagmus, slowing saccades, dysarthric speech, truncal and limb ataxia, decreased upper limb reflexes, and absent patellar and Achilles reflexes were noted. On the SARA scale (Schmitz-Hübsch et al*.*, 2006), he scored 10 points. Vibration sense was impaired distally on the lower limbs, but light touch, pinprick sensation, and proprioception were intact. Additionally, single choreiform movements of the upper limbs were observed.

The family history was unremarkable with no evidence of consanguinity, but the patient’s parents come from the same town. The pedigree of the family is shown in Fig. 1.

In the laboratory test, the AFP level was elevated up to 83.1 ng/ml (normal range 0.0–8.0 ng/ml). Nerve conduction studies showed axonal sensorimotor polyneuropathy. Visual evoked potentials (VEPs), brainstem auditory evoked potentials (BAEPs), and electroencephalogram EEG were normal. Brain MRI revealed marked atrophy of the vermis and cerebellar hemispheres. The supratentorial structures were unaffected (Fig. 2d–f). Neuropsychological evaluation revealed mild impairment of visual perception with preservation of other intellectual abilities.

### Genetic analysis

#### Patient 1

Based on the pedigree analysis, an autosomal recessive mode of inheritance was considered as well as an autosomal dominant de novo variant. Based on the patient’s phenotype, molecular tests for Friedreich ataxia, spinocerebellar ataxia type 1, 2, 3, and 8, and the most common mutations in mitochondrial DNA were performed on DNA isolated from peripheral blood. Genetic analysis of the DNA isolated from muscle tissue confirmed multiple deletions of mitochondrial DNA.

Since, in Poland, mutations in *POLG* are a frequent cause of mitochondrial disease accompanied by ataxia (Piekutowska-Abramczuk et al. 2019), the analysis of the *POLG* gene (NG_008218.2) based on a PCR test for most frequent *POLG* pathogenic variants followed by coding sequence analysis confirmed the presence of the heterozygous recessive p.Trp748Ser variant only, insufficient to cause the disease (Piekutowska-Abramczuk et al. 2019, patient P25). Therefore, whole exome sequencing analysis was performed.

Beside confirming the presence of the *POLG* pathogenic variant p.Trp748Ser, in-depth analysis of NGS data did not reveal any additional probably pathogenic variants in that gene, although *POLG* variants were excluded as a cause of the disease at that stage.

**Fig. 4:**
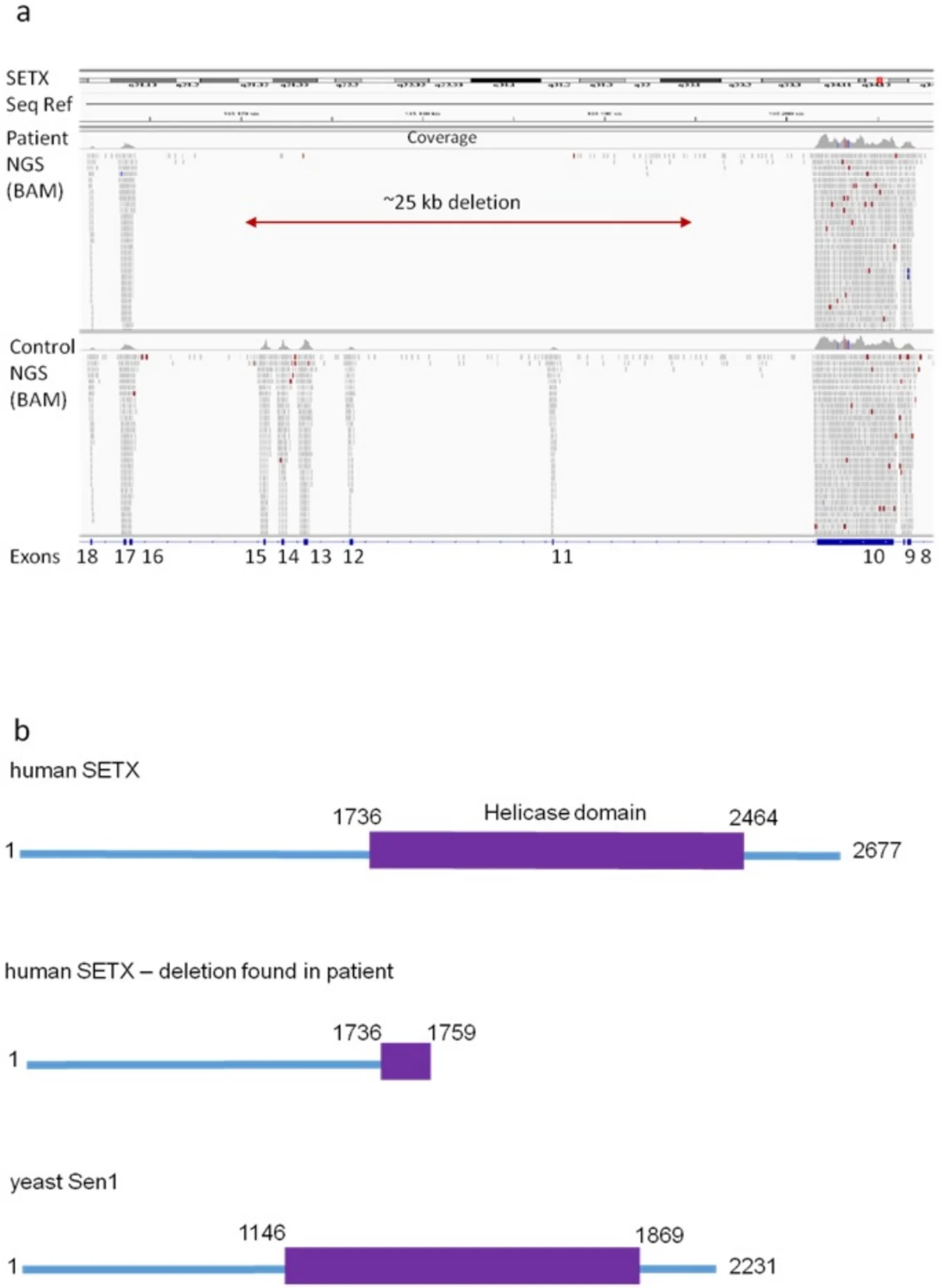
Integrative Genomics Viewer (IGV, Broad Institute) visualization of a fragment of the *SETX* gene on chromosome 9 encompassing the Patient 1 sequence compared to the control’s sequence. The top panel shows a chromosome ideogram with a red box indicating the section of the chromosome being displayed. The ruler reflects the visible portion of the chromosome. Tick marks indicate chromosome locations. The middle panel shows depth and read tracks of patient’s and control’s NGS data (BAM). The bottom panel corresponds to the *SETX* gene structure. Exons are highlighted by colored boxes **a**. Comparison of human senataxin, senataxin with a deletion found in Patient 1 and yeast Sen1. Numbers indicate amino acid residues. The conserved helicase domain is highlighted by colored boxes **b**

As the basic algorithm used for WES result analysis does not reveal CNVs, the HMZDelFinder algorithm was used. The HMZDelFinder identified one putative homozygous deletion chr9:132,295,852–132,311,876 encompassing 5 of 33 exons (exons 11–15 ref. NM_015046.7) of the *SETX* gene (Fig. 4) resulting in a premature stop codon and protein truncation p.Val1759GlufsTer5 (later called 11–15 deletion). The identified deletion was absent in gnomAD, Decipher, and ESP.

The fragment encompassing the region of deletion in the *SETX* gene was amplified by PCR (c.5275–1235 to c.6107–3435), and the presence of the detected variant was confirmed by Sanger sequencing. The lack of the correct allele was confirmed by PCR directed to the area within the deletion (c.5949–609 to c.6107 + 167).

The presence of the deletion was studied in healthy family members (parents, siblings) by PCR and Sanger sequencing. The deletion was present in the proband’s parents (II:3, II:4) and brother (III:3) in the heterozygous form.

#### Patient 2

As the first tests, triplet expansion in the genes causing Friedreich ataxia (FXN) and spinocerebellar ataxia type 2 (ATXN2) was excluded.

Later, the NGS panel of 152 genes correlated with ataxias, hereditary spastic paraplegias, and rare syndromes accompanied by ataxia or spastic paraplegia gave negative results. It was followed by WES analysis, which showed a homozygous deletion encompassing exons 11–15 of the *SETX* gene. The presence of the deletion was confirmed by MLPA. The presence of the deletion in heterozygous form was also confirmed in the patient’s parents by MLPA.

Analysis of NGS data from Medical University of Warsaw and Medgen Medical Center has revealed few heterozygous carriers of the 11–15 deletion (frequency 0.15% based on approximately 6000 samples) (Fig. 4).

## Discussion

In this study, we present two cases of ataxia and oculomotor apraxia type 2 (AOA2) caused by a potentially pathogenic homozygous variant of the *SETX* gene (NG_007946.1), a large deletion of 5 exons (exons 11–15; c.5275-1072_6107-3547del, ref. NM_015046.7) resulting in a premature stop codon and protein truncation p.Val1759GlufsTer5. In 2023, a deletion of exons 11–15 was reported in ClinVar as pathogenic (Variation ID: 3245202) and correlated with AOA2 and/or ALS4, although no clinical characteristics or phenotypic description of the affected individual was specified. In this article, we have determined the borders of the deletion and, most importantly, described and discussed phenotype—genotype correlation of patients with exon 11–15 deletion of the *SETX* gene. High-quality information about phenotype—genotype relationships is crucial for the diagnostic process, especially for adult onset neuromuscular disorders.

Pathogenic *SETX* variants include single nucleotide variants (SNV) with different consequences in protein sequences (missense, nonsense, splice site), frameshift variants, as well as deletions of various sizes. In the ClinVar database, 46 frameshift deletions are reported; most of them are pathogenic or likely pathogenic. Some large deletions were described by Nanetti and collaborators, e.g., a heterozygous (Kinkar et al. 2021) and homozygous exon 6 deletion, a heterozygous exon 8–10 deletion, and a homozygous exon 16–23 deletion (Nanetti et al. 2013); however, the exact breakpoints of all variants are unknown. Western blot analysis showed the virtual absence of senataxin in total lysate extracted from lymphoblastoid cell lines with *SETX* deletions (Nanetti et al. 2013) confirming the diagnosis of AOA2. Although mRNA studies and functional assays like described in Gaweda-Walerych et al. (2022) are needed for final confirmation of the molecular pathogenic mechanism, the molecular similarity of the identified variant to previously reported pathogenic alterations suggests a likely comparable background (Bernard et al. 2009).

Large scale deletions are also described in the ClinVar database. Reported as pathogenic are as follows: deletion of exons 3–10 (Variation ID: 3245203), 7–10 (Variation ID: 1459833), and 11–15 (Variation ID: 3245202); as likely pathogenic: deletion of exons 12–15 (Variation ID: 1705107) and as VUS: deletion of exon 7 (Variation ID: 1447311). Genotype–phenotype correlations were not provided in these reports. The majority of variants were broadly described as associated with *SETX*-related conditions or with AOA2/ALS4.

In the patients described here, the clinical phenotype aligns with that observed in previously reported individuals carrying biallelic structural variants of the *SETX* gene. Bernard et al. (2009) documented four AOA2 patients with gross copy number variants in *SETX* with early-onset cerebellar ataxia, cerebellar atrophy, peripheral sensorimotor neuropathy, oculomotor apraxia, dystonic posturing, and elevated α-fetoprotein. Conversely, cases with point variants in the *SETX* gene (missense, nonsense, splice‑site, or frameshift) often display a broadly similar core phenotype—progressive cerebellar ataxia, neuropathy, and elevated AFP (Chen et al. 2022). Some genotype–phenotype studies suggest that missense variants located within the C-terminal helicase domain may be associated with a milder phenotype, featuring later onset, less frequent oculomotor apraxia, and preserved ambulation longer into the disease course (Chen et al. 2022).

The presented patients’ history and results of additional studies are consistent with those described in the literature. AOA2 is one of the most common ataxias with an autosomal recessive mode of inheritance (Szpisjak et al. 2016). Brain neuroradiological studies revealed mainly cerebellar atrophy. One of the most characteristic laboratory findings is an elevated serum alpha-fetoprotein level. Another is axonal sensorimotor peripheral neuropathy. According to the literature data, when Friedreich’s ataxia is excluded, an elevated alpha-fetoprotein level is the indication for genetic testing for ataxia telangiectasia (AT) followed by AOA2. Nevertheless, the absence of telangiectasia, lack of immunodeficiency, and no malignancies favor the diagnosis of AOA2 (Brugger et al. 2014; Tariq et al. 2018). Some authors postulate that a young age of onset of ataxia and elevated serum AFP indicates the *SETX* gene should be analyzed (Choudry et al. 2018). Furthermore, due to signs of polyneuropathy, analysis of the *SETX* gene should be included in the neuropathy gene panel (Choudry et al. 2018). The described patients had no cardiological abnormalities, which also helps to distinguish this form of ataxia from Friedreich’s ataxia. Another form of neurodegenerative disorder caused by the *SETX* gene mutation is amyotrophic lateral sclerosis 4 (Duquette et al. 2005). However, this rare form of autosomal dominant ALS is characterized by an early onset and involvement of upper and lower motor neurons and lack of any sensory deficits (Anheim et al. 2009; Tariq et al. 2018). Analysis of medical data of 90 patients with AOA2 indicated that elevated AFP and cerebellar atrophy remain stable during the disease. Despite numerous studies, the exact cause of elevated AFP in AT and AOA2 is not known (Anheim et al. 2009). One of the theories involves abnormalities of polynucleotide unwinding in the liver of AOA2 patients, which change AFP gene transcription.

The origin of mtDNA deletions in Patient 1 remains unsolved. Although studies on yeast have shown that senataxin may be involved in mitochondrial function, a mitochondrial role and primarily mitochondrial localization of mammalian senataxin have not been confirmed so far (Sarikiet al. 2016). Senataxin depletion may lead to mtDNA R-loop accumulation, which is similar to the effect of the absence of RNAseH1 (Renaudin et al. 2021). While *RNASEH1* pathogenic variants lead to autosomal recessive mitochondrial disease with multiple mtDNA deletions, it seems probable that, at least in some cases, a *SETX* defect may give a similar effect. In the case of Patient 1, the explanation may be the synergistic effect of senataxin minor involvement in mitochondrial function together with residual *POLG* dysfunction caused by the heterozygous p.Trp748Ser pathogenic variant.

Altogether, taking into account pedigree analysis, the clinical picture, laboratory, and molecular results, we can conclude that the *SETX* 11–15 exons deletion is responsible for AOA2 in both cases. Although the patients carry a large deletion putatively inactivating senataxin, deleting almost the whole helicase domain, it does not lead to a more severe phenotype.

The existence of a homozygous, unique pathogenic variant may indicate consanguinity. In both cases, the family study does not confirm that the parents of the probands are related, although, in each case, they do come from the same region. Statistical analysis of NGS data from the Polish population identified few heterozygous carriers (frequency 0.15%) which may suggest its region-specific origin.

The diagnosis of neurodegenerative diseases remains a significant challenge due to the complex genotype–phenotype correlations. The severity and spectrum of clinical manifestations can vary widely, which often influences the choice of diagnostic approach. Given the genetic heterogeneity of these disorders, there is currently no universal genetic test that can reliably and rapidly lead to a definitive diagnosis. Therefore, targeted diagnostic strategies focusing on specific genes or gene panels are often required. Comprehensive analysis should include single nucleotide variants, CNVs, as well as repeat expansions. WES is currently the most commonly used method; however, it has important limitations, such as its inability to detect deep intronic variants, accurately assess repeat expansions, or identify mtDNA deletions. In some cases, a patient’s phenotype may result from more than one genetic disorder. Moreover, the potential interaction between multiple pathogenic variants should also be considered. This is exemplified by Patient 1, in whom a homozygous deletion in the *SETX* gene explains the neurological symptoms but does not directly account for the presence of mtDNA deletions. These deletions may result from *POLG* haploinsufficiency due to the presence of a pathogenic variant or from a combined effect of *POLG* and *SETX* dysfunctions, suggesting a possible interaction between these genes in the maintenance of mitochondrial DNA. Given that research on mitochondrial R-loops is still ongoing (Holt 2022), it cannot be excluded that *SETX* plays a role in this process. Its dysfunction may disturb mtDNA metabolism and contribute to the formation of mtDNA deletions.

## Data Availability

The data that support the findings of this study are available from the corresponding author upon reasonable request.

## References

[CR1] Aguilera A, García-Muse T (2012) R loops: from transcription byproducts to threats to genome stability. Mol Cell 46:115–124. 10.1016/j.molcel.2012.04.009 22541554

[CR2] Aguilera A, García-Muse T (2013) Causes of genome instability. Ann Rev Genet 47:1–32. 10.1146/annurev-genet-111212-133232 23909437

[CR3] Aguilera A, Gómez-González B (2008) Genome instability: a mechanistic view of its causes and consequences. Nat Rev Genet 9:204–217. 10.1038/nrg2268 18227811

[CR4] Algahtani H, Shirah B, Algahtani R et al (2018) Ataxia with ocular apraxia type 2 not responding to 4-aminopyridine: a rare mutation in the *SETX* gene in a Saudi patient. Intract Rare Dis Res 7:275–279. 10.5582/irdr.2018.01107 PMC6290838 30560021

[CR5] Alzu A, Bermejo R, Begnis M et al (2012) Senataxin associates with replication forks to protect fork integrity across RNA-polymerase-II-transcribed genes. Cell 151:835–846. 10.1016/j.cell.2012.09.041 23141540 PMC3494831

[CR6] Anheim M, Monga B, Fleury M et al (2009) Ataxia with oculomotor apraxia type 2: clinical, biological and geno-type/phenotype correlation study of a cohort of 90 patients. Brain 132:2688–2698. 10.1093/brain/awp211 19696032

[CR7] Arning L, Epplen JT, Rahikkala E et al (2013) The *SETX* missense variation spectrum as evaluated in patients with ALS4-like motor neuron diseases. Neurogenetics 14:53–61. 10.1007/s10048-012-0347-4 23129421

[CR8] Becherel OJ, Yeo AJ, Stellati A et al (2013) Senataxin plays an essential role with DNA damage response proteins in meiotic recombination and gene silencing. PLoS Genet 9:e1003435. 10.1371/journal.pgen.1003435 23593030 PMC3623790

[CR9] Becherel OJ, Fogel BL, Zeitlin SI et al (2019) Disruption of spermatogenesis and infertility in ataxia with oculomotor apraxia type 2 AOA2. Cerebellum 18:448–456. 10.1007/s12311-019-01012-w 30778901 PMC6520128

[CR10] Bernard V, Minnerop M, Bürk K et al (2009) Exon deletions and intragenic insertions are not rare in ataxia with oculomotor apraxia 2. BMC Med Genet 10:87. 10.1186/1471-2350-10-87 19744353 PMC2749023

[CR11] Brugger F, Schüpbach M, Koenig M et al (2014) The clinical spectrum of ataxia with oculomotor apraxia type 2. Mov Disord Clin Pract 1:106–109. 10.1002/mdc3.12021 30363866 PMC6183195

[CR12] Catford SR, O’Bryan MK, McLachlan RI et al (2019) Germ cell arrest associated with a *SETX* mutation in ataxia oculomotor apraxia type 2. Reprod Biomed Online 38:961–965. 10.1016/j.rbmo.2018.12.042 30642639

[CR13] Chen S, Du J, Jiang H et al (2022) Ataxia oculomotor apraxia type 2 caused by a novel homozygous mutation in SETX gene, and literature review. Front Mol Neurosci 15:1019974. 10.3389/fnmol.2022.1019974 36438189 PMC9684320

[CR14] Chen X, Müller U, Sundling KE, Brow DA (2014) *Saccharomyces cerevisiae* Sen1 as a model for the study of mutations in human senataxin that elicit cerebellar ataxia. Genetics 198:577–590. 10.1534/genetics.114.167585 25116135 PMC4196614

[CR15] Chen Y-Z, Bennett CL, Huynh HM et al (2004) DNA/RNA helicase gene mutations in a form of juvenile amyotrophic lateral sclerosis ALS4. Am J Hum Genet 74:1128–1135. 10.1086/421054 15106121 PMC1182077

[CR16] Choudry TN, Hilton-Jones D, Lennox G, Houlden H (2018) Ataxia with oculomotor apraxia type 2: an evolving axonal neu-ropathy. Pract Neurol 18:52–56. 10.1136/practneurol-2017-001711 29212862

[CR17] Cohen S, Puget N, Lin Y-L et al (2018) Senataxin resolves RNA:DNA hybrids forming at DNA double-strand breaks to prevent translocations. Nat Commun 9:1–14. 10.1038/s41467-018-02894-w 29416069 PMC5803260

[CR18] Criscuolo C, Chessa L, Giandomenico SD et al (2006) Ataxia with oculomotor apraxia type 2: a clinical, pathologic, and genetic study. Neurology 66:1207–1210. 10.1212/01.wnl.0000208402.10512.4a 16636238

[CR19] De Amicis A, Piane M, Ferrari F et al (2011) Role of senataxin in DNA damage and telomeric stability. DNA Repair 10:199–209. 10.1016/j.dnarep.2010.10.012 21112256

[CR20] Duquette A, Roddier K, McNabb-Baltar J et al (2005) Mutations in senataxin responsible for Quebec cluster of ataxia with neuropathy. Ann Neurol 57:408–414. 10.1002/ana.20408 15732101

[CR21] Fogel BL, Perlman S (2006) Novel mutations in the senataxin DNA/RNA helicase domain in ataxia with oculomotor apraxia 2. Neurology 67:2083–2084. 10.1212/01.wnl.0000247661.19601.28 17159128

[CR22] Fogel BL, Lee JY, Lane J et al (2012) Mutations in rare ataxia genes are uncommon causes of sporadic cerebellar ataxia. Mov Disord 27:442–446. 10.1002/mds.24064 22287014 PMC3323119

[CR23] Gambin T, Akdemir ZC, Yuan B et al (2017) Homozygous and hemizygous CNV detection from exome sequencing data in a Mendelian disease cohort. Nucleic Acids Res 45:1633–1648. 10.1093/nar/gkw1237 27980096 PMC5389578

[CR24] García-Muse T, Aguilera A (2016) Transcription–replication conflicts: how they occur and how they are resolved. Nat Rev Mol Cell Biol 17:553–563. 10.1038/nrm.2016.88 27435505

[CR25] Gatti V, De Domenico S, Melino G et al (2023) Senataxin and R-loops homeostasis: multifacedimplications in carcinogenesis. Cell Death Discov 9:145. 10.1038/s41420-023-01441-x 37147318 PMC10163015

[CR26] Gaweda-Walerych K, Sitek EJ, Borczyk M et al (2022) Patient with corticobasal syndrome and progressive non-fluent aphasia (CBS-PNFA), with variants in *ATP7B*, *SETX*, *SORL1*, and *FOXP1* genes. Genes (Basel) 13:2361. 10.3390/genes13122361 36553628 PMC9778325

[CR27] Gazulla J, Benavente I, López-Fraile IP et al (2009) Sensorimotor neuronopathy in ataxia with oculomotor apraxia type 2. Muscle Nerve 40:481–485. 10.1002/mus.21328 19618424

[CR28] Groh M, Gromak N (2014) Out of balance: R-loops in human disease. PLoS Genet 10:e1004630. 10.1371/journal.pgen.1004630 25233079 PMC4169248

[CR29] Groh M, Albulescu LO, Cristini A, Gromak N (2017) Senataxin: genome guardian at the interface of transcription and neurodegeneration. J Mol Biol 429:3181–3195. 10.1016/j.jmb.2016.10.021 27771483

[CR30] Grunseich C, Wang IX, Watts JA et al (2018) Senataxin mutation reveals how R-loops promote transcription by blocking DNA methylation at gene promoters. Mol Cell 69:426–437. 10.1016/j.molcel.2017.12.030 29395064 PMC5815878

[CR31] Grzechnik P, Gdula MR, Proudfoot NJ (2015) Pcf11 orchestrates transcription termination pathways in yeast. Genes Dev 29:849–861. 10.1016/j.molcel.2015.01.011 25877920 PMC4403260

[CR32] Hamperl S, Cimprich KA (2014) The contribution of co-transcriptional RNA:DNA hybrid structures to DNA damage and genome instability. DNA Repair 19:84–94. 10.1016/j.dnarep.2014.03.023 24746923 PMC4051866

[CR33] Hasanova Z, Klapstova V, Porrua O, Stefl R, Sebesta M (2023) Human senataxin is a bona fide R-loop resolving enzyme and transcription termination factor. Nucleic Acids Res 51(6):2818–2837. 10.1093/nar/gkad092 36864660 PMC10085699

[CR34] Hatchi E, Skourti-Stathaki K, Ventz S et al (2015) *BRCA1* recruitment to transcriptional pause sites is required for R-loop-driven DNA damage repair. Mol Cell 57:636–647. 10.1016/j.molcel.2015.01.011 25699710 PMC4351672

[CR35] Holt IJ (2022) R-loops and mitochondrial DNA metabolism. Methods Mol Biol 2528:173–202. 10.1007/978-1-0716-2477-7_12 35704192

[CR36] Homer HA (2021) Senataxin: a new guardian of the female germline important for delaying ovarian aging. Front Genet 12:647996. 10.3389/fgene.2021.647996 33995483 PMC8118517

[CR37] Ichikawa Y, Ishiura H, Mitsui J et al (2013) Exome analysis reveals a Japanese family with spinocerebellar ataxia, autosomal recessive 1. J Neurol Sci 331:158–160. 10.1016/j.jns.2013.05.018 23786967

[CR38] Kaliszewska M, Kruszewski J, Kierdaszuk B et al (2015) Yeast model analysis of novel polymerase gamma variants found in patients with autosomal recessive mitochondrial disease. Hum Genet 134:951–966. 10.1007/s00439-015-1578-x 26077851 PMC4529462

[CR39] Kierdaszuk B, Jamrozik Z, Tońska K et al (2009) Mitochondrial cytopathies: clinical, morphological and genetic character-istics. Neurol Neurochir Pol 43:216–227 19618304

[CR40] Kinkar JS, Jameel PZ, Kumawat BL et al (2021) Heterozygous deletion in exon 6 of *STEX* gene causing ataxia with oculomotor apraxia type 2 (AOA-2) with ovarian failure. BMJ Case Reports CP 14:e241767. 10.1136/bcr-2021-241767 PMC8246282 34193451

[CR41] Lavin MF, Yeo AJ, Becherel OJ (2013) Senataxin protects the genome. Rare Dis 1:e25230. 10.4161/rdis.25230 25003001 PMC3927485

[CR42] Le Ber I, Brice A, Dürr A (2005) New autosomal recessive cerebellar ataxias with oculomotor apraxia. Curr Neurol Neurosci Rep 5:411–417. 10.1007/s11910-005-0066-4 16131425

[CR43] Lynch DR, Braastad CD, Nagan N (2007) Ovarian failure in ataxia with oculomotor apraxia type 2. Am J Med Genet A 143A:1775–1777. 10.1002/ajmg.a.31816 17593543

[CR44] Makharashvili N, Arora S, Yin Y et al (2018) Sae2/CtIP prevents R-loop accumulation in eukaryotic cells. Elife 7:e42733. 10.7554/eLife.42733 30523780 PMC6296784

[CR45] Marelli C, Guissart C, Hubsch C et al (2016) Mini-exome coupled to read-depth based copy number variation analysis in patients with inherited ataxias. Hum Mutat 37:1340–1353. 10.1002/humu.23063 27528516

[CR46] Miller MS, Rialdi A, Ho JSY et al (2015) Senataxin suppresses the antiviral transcriptional response and controls viral biogenesis. Nat Immunol 16:485. 10.1038/ni.3132 25822250 PMC4406851

[CR47] Moreira M-C, Klur S, Watanabe M et al (2004) Senataxin, the ortholog of a yeast RNA helicase, is mutant in ataxia-ocular apraxia 2. Nat Genet 36:225–227. 10.1038/ng1303 14770181

[CR48] Nanetti L, Cavalieri S, Pensato V et al (2013) *SETX* mutations are a frequent genetic cause of juvenile and adult onset cere-bellar ataxia with neuropathy and elevated serum alpha-fetoprotein. Orphanet J Rare Dis 8:123. 10.1186/1750-1172-8-123 23941260 PMC3751478

[CR49] Nicolaou P, Georghiou A, Votsi C et al (2008) A novel c.5308_5311delGAGA mutation in senataxin in a Cypriot family with an autosomal recessive cerebellar ataxia. BMC Med Genet 9:28. 10.1186/1471-2350-9-28 18405395 PMC2330029

[CR51] Piekutowska-Abramczuk D, Kaliszewska M, Sułek A et al (2019) The frequency of mitochondrial polymerase gamma related disorders in a large Polish population cohort. Mitochondrion 47:179–187. 10.1016/j.mito.2018.11.004 30423451

[CR52] Ploski R, Pollak A, Müller S et al (2014) Does p. Q247X in TRIM63 cause human hypertrophic cardiomyopathy? Circ Res 114:e2–e5. 10.1161/CIRCRESAHA.114.302662 24436435

[CR53] Renaudin X, Lee M, Shehata M, Surmann EM, Venkitaraman AR (2021) *BRCA2* deficiency reveals that oxidative stress impairs *RNaseH1* function to cripple mitochondrial DNA maintenance. Cell Rep 36(5):109478. 10.1016/j.celrep.2021.109478 34348152 PMC8356021

[CR54] Sariki SK, Sahu PK, Golla U et al (2016) Sen1, the homolog of human senataxin, is critical for cell survival through regulation of redox homeostasis, mitochondrial function, and the TOR pathway in *Saccharomyces cerevisiae*. FEBS J 283:4056–4083. 10.1111/febs.13917 27718307

[CR55] Schmitz-Hübsch T, duMontcel ST, Baliko L, Berciano J, Boesch S, Depondt C, Giunti P, Globas C, Infante J, Kang JS, Kremer B, Mariotti C, Melegh B, Pandolfo M, Rakowicz M, Ribai P, Rola R, Schöls L, Szymanski S, van de Warrenburg BP, Dürr A, Klockgether T, Fancellu R (2006) Scale for the assessment and rating of ataxia: development of a newclinicalscale. Neurology 66(11):1717–1720. 10.1212/01.wnl.0000219042.60538.92 16769946

[CR56] Skourti-Stathaki K, Proudfoot NJ, Gromak N (2011) Human senataxin resolves RNA/DNA hybrids formed at transcriptional pause sites to promote Xrn2-dependent termination. Mol Cell 42:794–805. 10.1016/j.molcel.2011.04.026 21700224 PMC3145960

[CR57] Suraweera A, Becherel OJ, Chen P et al (2007) Senataxin, defective in ataxia oculomotor apraxia type 2, is involved in the defense against oxidative DNA damage. J Cell Biol 177:969–979. 10.1083/jcb.200701042 17562789 PMC2064358

[CR59] Szpisjak L, Obal I, Engelhardt JI et al (2016) A novel *SETX* gene mutation producing ataxia with oculomotor apraxia type 2. Acta Neurol Belg 116:405–407. 10.1007/s13760-015-0569-y 26811093

[CR60] Tariq H, Imran R, Naz S (2018) A novel homozygous variant of SETX causes ataxia with oculomotor apraxia type 2. J Clin Neurol 14:498–504. 10.3988/jcn.2018.14.4.498 30198223 PMC6172491

[CR62] Yüce Ö, West SC (2013) Senataxin, defective in the neurodegenerative disorder ataxia with oculomotor apraxia 2, lies at the interface of transcription and the DNA damage response. Mol Cell Biol 33:406–417. 10.1128/MCB.01195-12 23149945 PMC3554130

